# The Prp19 Complex Directly Functions in Mitotic Spindle Assembly

**DOI:** 10.1371/journal.pone.0074851

**Published:** 2013-09-19

**Authors:** Jennifer C. Hofmann, Justus Tegha-Dunghu, Stefanie Dräger, Cindy L. Will, Reinhard Lührmann, Oliver J. Gruss

**Affiliations:** 1 Zentrum für Molekulare Biologie der Universität Heidelberg (ZMBH), DKFZ-ZMBH Alliance, Heidelberg, Germany; 2 Department of Cellular Biochemistry, Max Planck Institute for Biophysical Chemistry, Göttingen, Germany; Institut de Génétique et Développement de Rennes, France

## Abstract

The conserved Prp19 (pre-RNA processing 19) complex is required for pre-mRNA splicing in eukaryotic nuclei. Recent RNAi screens indicated that knockdown of Prp19 complex subunits strongly delays cell proliferation. Here we show that knockdown of the smallest subunit, BCAS2/Spf27, destabilizes the entire complex and leads to specific mitotic defects in human cells. These could result from splicing failures in interphase or reflect a direct function of the complex in open mitosis. Using *Xenopus* extracts, in which cell cycle progression and spindle formation can be reconstituted in vitro, we tested Prp19 complex functions during a complete cell cycle and directly in open mitosis. Strikingly, immunodepletion of the complex either before or after interphase significantly reduces the number of intact spindles, and increases the percentage of spindles with lower microtubule density and impaired metaphase alignment of chromosomes. Our data identify the Prp19 complex as the first spliceosome subcomplex that directly contributes to mitosis in vertebrates independently of its function in interphase.

## Introduction

To enable spindle formation, microtubules dramatically change their dynamics and organization at the transition from interphase to mitosis. Global changes in microtubule architecture are primarily a consequence of altered patterns of microtubule associated and microtubule motor proteins whose activity is regulated by cell cycle-dependent expression, posttranslational modifications and relocalisation from the breaking down nucleus in higher eukaryotes (open mitosis) [1-5] [6] [7-10]. TPX2, for instance, accumulates in the interphase nucleus during S and G2 phase but fulfills its essential function in mitotic spindle assembly in the M-phase cytoplasm after Nuclear Envelope Breakdown (NEB) [11] [12]. The nuclear intermediate filament protein lamin B constitutes a spindle matrix in mitosis, supporting assembly and function of the microtubule-based spindle structure [13,14]. However, potential roles in cell division of most nuclear proteins, including proteins of the gene expression machinery involved in mRNA transcription and mRNA processing, remain largely unclear.

Comprehensive RNAi screens recently revealed compromised functions in cell proliferation after knockdown of proteins with established functions in splicing, in particular the components of the conserved Prp19 complex [15] [16,17] [18]. The Prp19 complex consists of 4 core subunits in humans, PRPF19, CDC5L, PLRG1 and SPF27/BCAS2, as well as 3 associated proteins AD002, CTNNBL1 and HSP73 [19] [20] [21].

Proliferation defects upon knocking down Prp19 complex proteins, or other gene products required for splicing, may be a result of changed patterns of mature mRNAs, and consequently their respective translation products. Qualitative or quantitative alterations in splicing of mRNAs encoding spindle proteins or kinetochore components that have to be synthesized de novo in every cell cycle will cause mitotic abnormalities [18]. Alternatively, proteins involved in splicing may have an additional, direct function in open mitosis.

Here we show that knockdown of Prp19 complex components in intact human cells leads to specific mitotic defects. Cells arrest at a prometaphase-like state due to chromosome alignment errors and defective microtubule to kinetochore interactions. In order to further analyze the function of the Prp19 complex in open mitosis, we employed *Xenopus* egg extracts, in which spindle assembly can be faithfully recapitulated [22]. In this system, specific immunodepletion of the Prp19 complex directly in mitosis leads to an overall lowered spindle assembly efficiency, and the formation of spindles with lower microtubule density and compromised chromosome alignment. Our data strongly support the idea of a direct, interphase-independent role of the Prp19 complex in open mitosis.

## Results and Discussion

**Figure 1:**
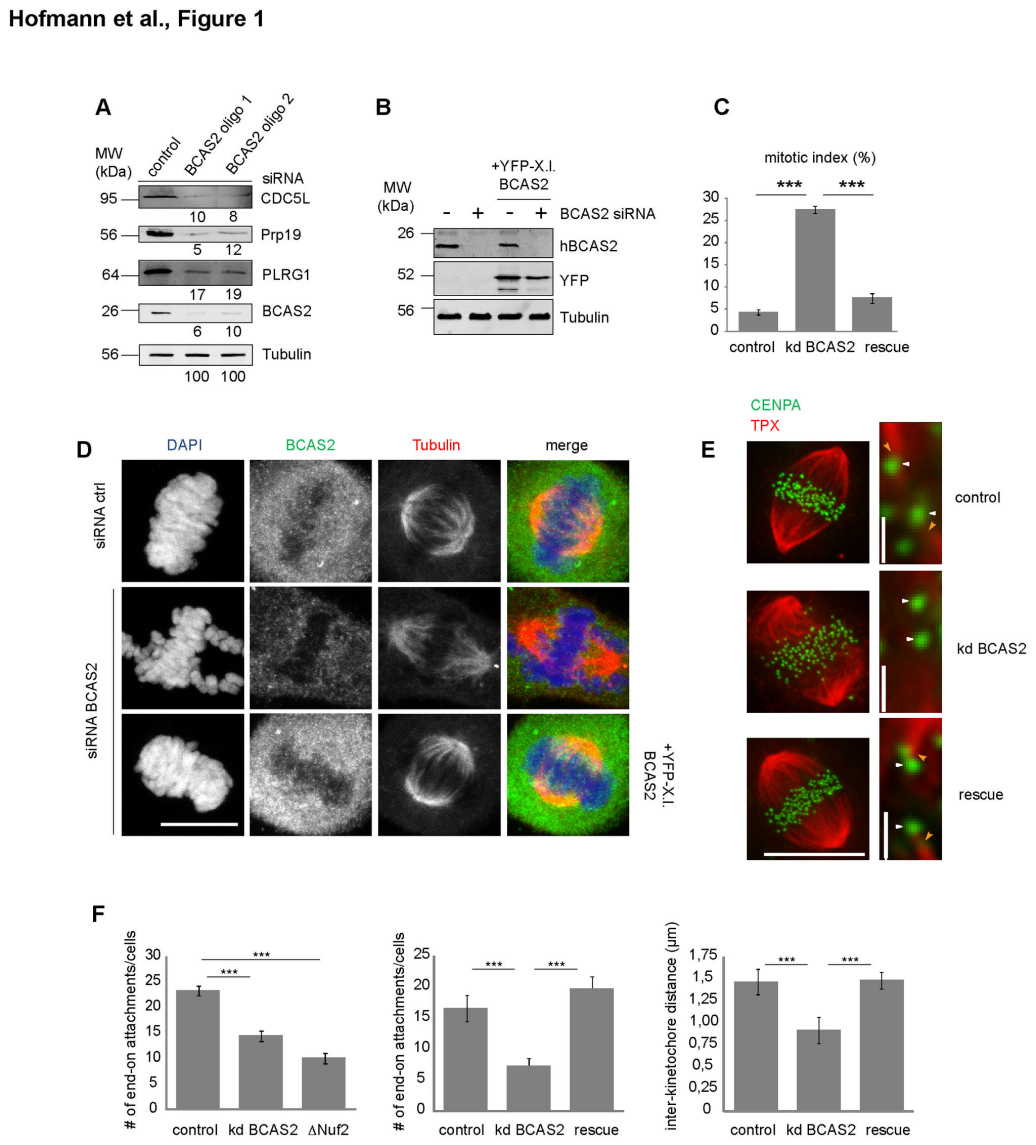
Knockdown of Prp19 complex components in human cells compromises mitosis. (A): HeLa cell lysates analyzed by immunoblotting with Prp19 complex antibodies, after knockdown of BCAS2 by siRNA (1 and 2). Numbers indicate protein levels relative to Tubulin. (B-E) BCAS2 (oligo 1) knockdown (siRNA BCAS2, kd BCAS2) and control (siRNA ctrl). YFP-Xenopus *laevis* (X.l.) BCAS2 was expressed to complement knockdown of the human (h.s.) BCAS2 (B): Immunoblot to show knockdown of endogenous human BCAS2 and re-expression of the *Xenopus* ortholog. (C) Quantification of mitotic indices determined from three independent experiments (N=500); graph shows mean values +/- s.e.m. Significance values were calculated using an unpaired two-tailed T-test. ***: P < 0.001. (D) BCAS2 expression and localization were analyzed by indirect immunofluorescence with BCAS2 (green in merge) and α-Tubulin (red in merge) antibodies. DAPI (blue in merge) was used to stain the DNA. (E and F) Cells after knockdown (kd) of BCAS2 (oligo 1), or control cells were immunostained with antibodies against the centromere protein CENPA (green) and the spindle protein TPX2 (red). Cells expressing X.l. YFP-BCAS2 were analyzed for the rescue situation. (E) Representative images. (F) the distance between white arrowheads (E) was quantified as interkinetochore distance; orange arrowheads (E) exemplify microtubule to kinetochore end-on attachments. Nuf2 knock-down cells served as a positive control. Quantifications were determined from three independent experiments (N=60); graph shows mean values +/- s.e.m. Significance values were calculated using an unpaired two-tailed T-test. ***: P < 0.001. Scalebars: 10 µm; 1 µm in magnifications in (E).

In order to characterize the quality and specificity of cell division defects after knockdown of Prp19 complex components, we analyzed HeLa cells depleted of BCAS2/Spf27, the smallest Prp19 core complex component. Reduction of BCAS2 by 90% or more (Figure 1A, BCAS2) led to downregulation of the other Prp19 complex core components CDC5L, PRPF19 and PLRG1 (Figure 1A), confirming their functional interaction. The knockdown diminished BCAS2 as well as PRPF19 and PLRG1 immunofluorescence signals in interchromatin spaces (Figure S1A and B [23], and data not shown) and decreased a dispersed BCAS2 signal in mitotic cells (Figure 1D). Importantly, BCAS knockdown yielded a mitotic index of up to 40% (Figure 1C). Knockdown cells accumulated in prometaphase (PM)- or metaphase (M) -like stages with impaired chromosome alignment indicative of defects in spindle function (Figure 1D). Time-lapse imaging of cells expressing Histone2B-eGFP after BCAS2 knockdown confirmed our observations: cells entered mitosis with condensed chromosomes but failed to stably line up all chromosomes in the metaphase plate (movies S1 and S2). Like endogenous human (h.s.) BCAS2, the *Xenopus laevis* (X.l.) ortholog localized in interchromatin spaces in interphase (Figure S1C and D, rescue) and dispersed throughout the mitotic cytoplasm (Figure 1D, rescue) when expressed as a YFP fusion. Expression of YFP-x.l.Bcas2 was unaffected by siRNA knockdown (Figure 1B, Figure S1) and rescued mitotic defects almost completely as judged by a reduced mitotic index (Figure 1C, rescue). Along with XLBcas2 re-expression in knock-down cells, the nuclear levels PRPF19 and PLRG1 were restored to endogenous levels (Figure S1D). This clearly demonstrates the specificity of the observed mitotic phenotype after knockdown of Prp19 complex components, and confirms that *Xenopus* Bcas2 is a bona fide ortholog of human BCAS2.

In order to further investigate the molecular basis for chromosome misalignment, we directly assayed functional, end-on attachment of microtubules to kinetochores using antibodies against TPX2 to visualize microtubules, and against CENPA to stain kinetochores (Figure 1E). Compared to controls, the number of end-on attachments per cell was significantly reduced in human BCAS2 knockdown cells (Figure 1E and F), reaching levels similar to those seen after direct knock-down of the kinetochore component Nuf2 (Figure 1F) but reverted to control levels after expression of functional *Xenopus* Bcas2 (Figure 1E and F, rescue). Functional end-on attachments generate tension at the microtubule kinetochore interface to separate the oppositely locating kinetochores of sister chromatids. Upon Bcas2 knockdown, inter-kinetochore distances were reduced to ca. 0.9 µm as compared to ca. 1.5 µm in control cells, or after rescue upon expression of *Xenopus* Bcas2 (Figure 1E and F). This clearly indicates that metaphase alignment problems after Prp19 complex knockdown result from compromised microtubule to kinetochore interactions. Taken together, siRNA-mediated knockdown of BCAS2, the smallest protein of the human PRPF19 complex, reduced BCAS2 levels and downregulated the other PRPF19 complex components PRPF19, PRLG1 and CDC5L. Under these conditions, the splicing of many gene products will be affected, in particular those that are produced de novo in every cell cycle. Our data therefore suggest that failure in metaphase alignment after BCAS2 knockdown may be a consequence of erroneous splicing of one or several factors required for spindle formation during preceding interphase. Alternatively, the Prp19 complex, or single complex component, might have a splicing-independent function in open mitosis despite the fact that no particular BCAS2 or PRPF19 localization could be observed in metaphase (M) cells (Figure 1D, Figure S1C and D).

However, dissecting the known functions of the Prp19 complex in splicing in the interphase nucleus from a hypothetical function in open mitosis was not readily feasible in human cells.

In unfertilized *Xenopus* eggs, proteins and mature mRNAs are stockpiled to ensure the fast cleavage divisions of the early embryo. Egg extracts proceed through the cell cycle even without endogenous mRNAs and even when protein translation is generally abolished. They only require synthesis of mitotic cyclin [22]. Sperm nuclei incubated under these conditions will replicate chromatin and duplicate the activated centrosome, which allows the assembly of a bipolar spindle that comprises two active centrosomes, replicated chromosomes, as well as duplicated kinetochores [24]. In this system, acute immunodepletion of proteins or protein complexes allows to test their function at any stage during the cell cycle and to experimentally uncouple a mitotic function from the known function of the Prp19 complex during interphase.

**Figure 2:**
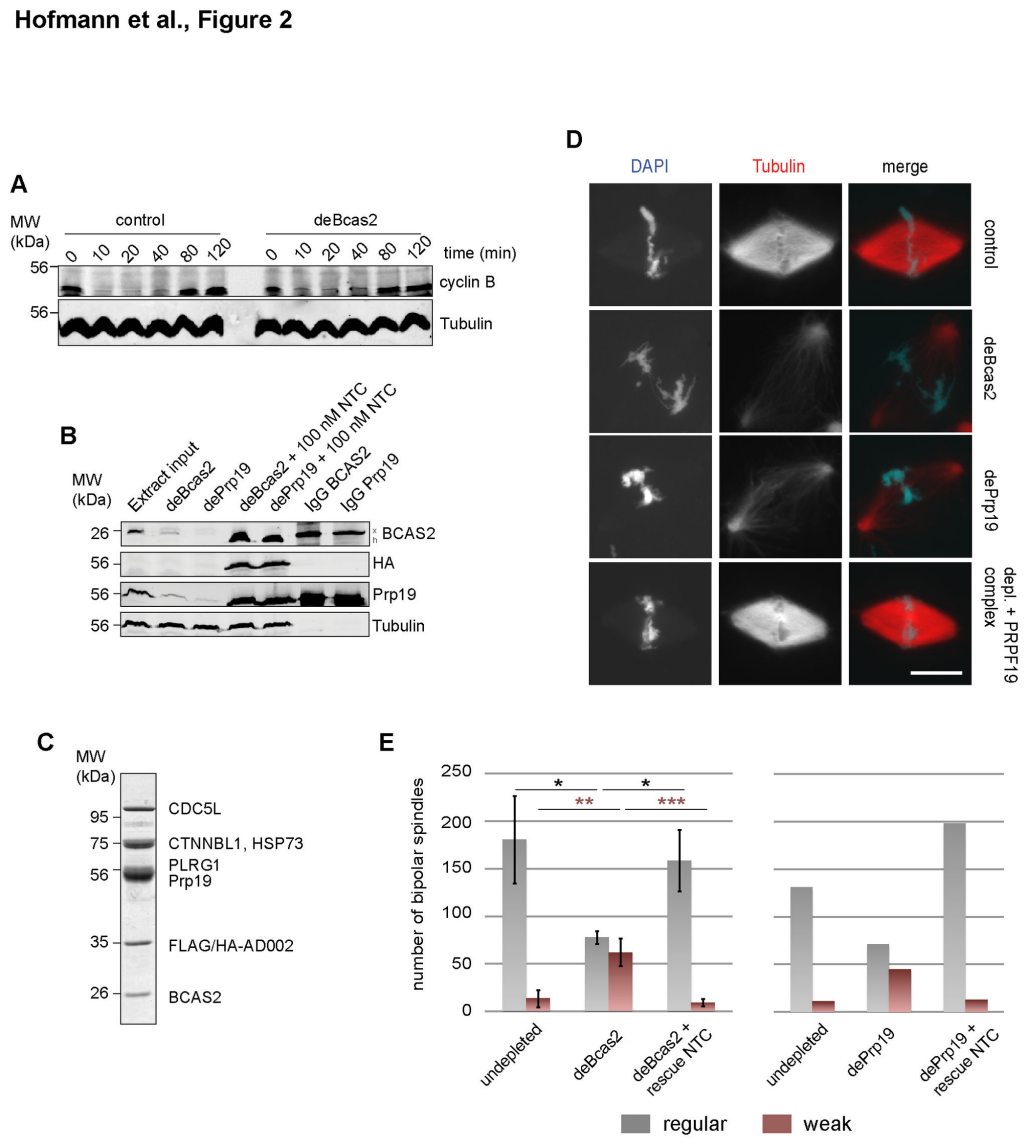
Depletion of the Prp19 complex disturbs spindle formation in *Xenopus* egg extracts. (A): Control *Xenopus* egg extracts or extracts after depletion of the Prp19 complex (deBCAS2) were released from metaphase (times after release are indicated) and the degradation and re-accumulation of cyclin B was monitored by immunoblot. Tubulin served as a loading control. (B): Extracts were depleted of the Prp19 complex by antibodies against BCAS2 (deBCAS2) or Prp19 (dePrp19) and complemented with 100 nM purified human Prp19 complex (see C, Coomassie staining). Extract samples, and the immunoglobulin beads after depletion, were analyzed by Immunoblot with *Xenopus* BCAS2 and Prp19 antibodies. Tubulin served as a loading control for the extract samples. (D): Spindle assembly was monitored in control, BCAS2 (deBCAS2), or Prp19 (dePrp19) depleted egg extracts, or in depleted extracts after re-addition of 100 nM Prp19 complex (see panel C) after a complete cell cycle in the presence of sperm nuclei (DAPI, DNA, blue in merge) and Cy3-tubulin (red in merge). Scalebars: 20 µm. (E): Quantification of normal (grey) and weak (red) spindles in control, depleted (dePrp19) and reconstituted extracts (rescue) spindles in 20 µl extract. The graph (left) shows values from three independent experiments +/- s.e.m. after depletion using BCAS2 antibodies, and from one representative experiment after depletion using Prp19 antibodies (right). Significance values (left) were calculated using an unpaired two-tailed T-test. *: P < 0.05, **: P < 0.01, ***: P < 0.001.

We first assayed for a function of the Prp19 complex in cell cycle progression and synthesis of mitotic cyclins in the in vitro cell cycle. Immunodepletion of the Prp19 complex (Figure 2B) did not affect cell cycle progression in *Xenopus* egg extracts, as indicated by normal re-accumulation of cyclin B (Figure 2A). We then tested spindle assembly after the loss of the Prp19 complex for one complete cell cycle in *Xenopus* extracts. To confirm the specificity of depletion, we used antibodies against Bcas2 as well as against *Xenopus* Prp19, which both efficiently depleted their direct antigen and the respective other Prp19 complex protein (Figure 2B). We then established conditions for a specific rescue of Prp19 complex depletion. As the entire Prp19 complex cannot be reconstituted from recombinant proteins, we affinity-purified the complex from human cells expressing a FLAG/HA tagged version of AD002. Besides BCAS2, PRPPF19, CDC5L and PLRG1, the purified human Prp19 complex also contained tagged AD0002, HSP73 and CTNNBL1 ( [21], Figure 2C).

Bipolar spindles still assembled in Bcas2-depleted extracts but the number of spindles was reduced by more than 50%. Moreover, a significantly higher number (3-10 fold) of spindles displayed reduced microtubule density in the vicinity of chromatin (Figure 2D and E). A qualitatively and quantitatively very similar phenotype was observed when we immunodepleted the Prp19 complex using antibodies against *Xenopus* Prp19 (Figure 2B, D and E). To complement extracts depleted of the Prp19 complex, we added back 100 nM of the human complex, which restored normal spindle assembly to near control levels (Figure 2C to E).

**Figure 3:**
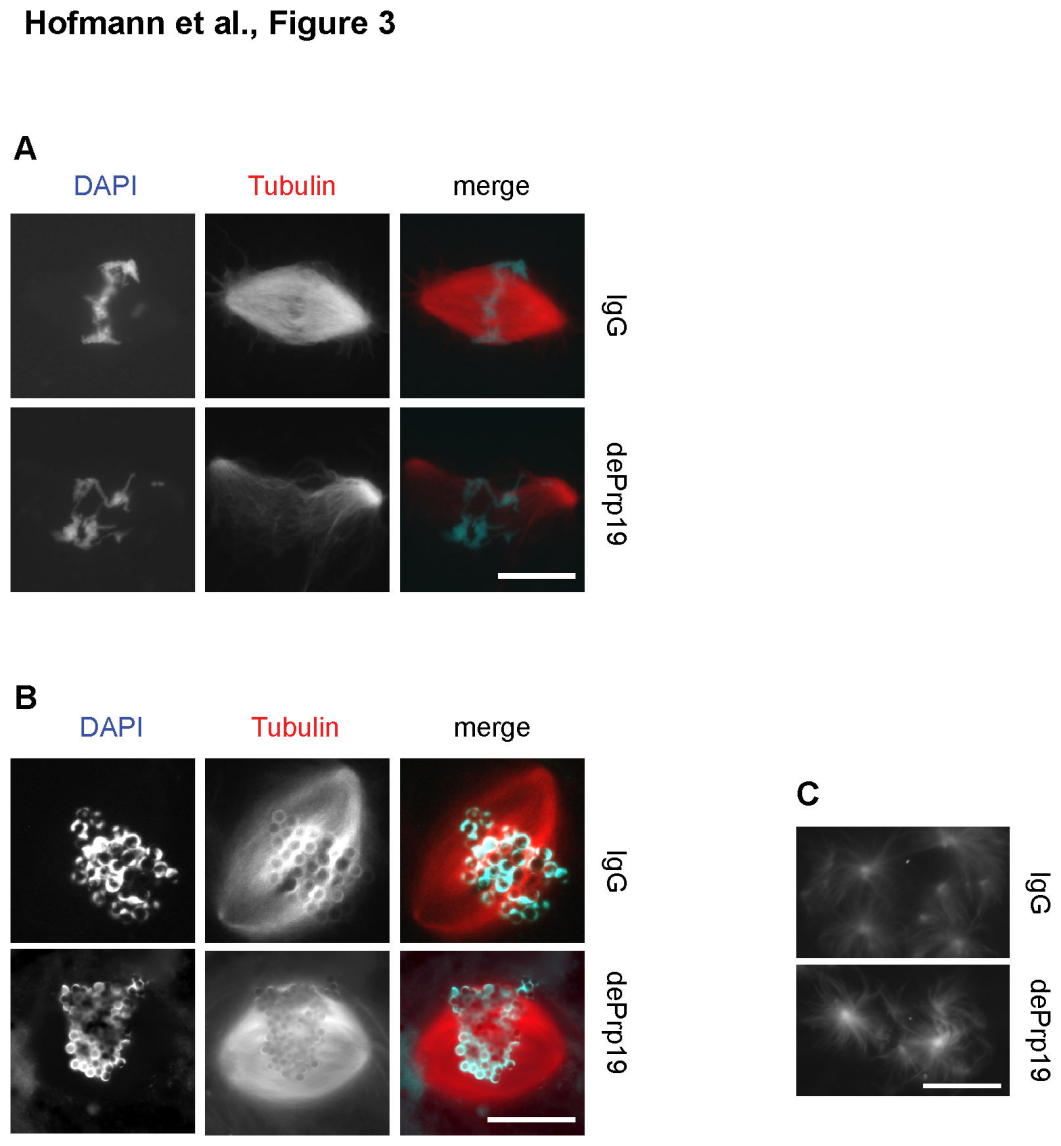
Immunodepletion of the Prp19 complex from *Xenopus* egg extracts does not compromise mitotic microtubule assembly in general. *Xenopus* egg extracts arrested in metaphase were depleted of the Prp19 complex using a specific antibody (dePrp19); an unspecific IgG served as a control. (A) Spindle assembly around sperm nuclei was monitored in *Xenopus* egg extracts after a complete cell cycle. (B): Spindle assembly around chromatin beads was monitored in *Xenopus* egg extracts after a complete cell cycle. (A) -(B): Microtubules are shown in red, chromatin (DAPI) in blue. (C): Microtubule aster assembly upon Ran GTP addition was monitored in cycled egg extracts. Scale bars: 20 µm.

Interestingly, the observed abnormalities in spindle assembly were not due to generally altered microtubule assembly activity in *Xenopus* egg extracts after Prp19 depletion. While reduced spindle assembly efficiency and weak spindles were observed after Prp19 complex depletion when spindles formed around cycled sperm nuclei (Figure 3A), spindles efficiently formed around chromatin beads [25] despite Prp19 complex depletion for a full cell cycle (Figure 3B). Similarly, aster-like structures assembled with the same efficiency in Prp19 complex depleted or control *Xenopus* egg extracts upon addition of Ran.GTP [26], which mimics the activity of chromatin (Figure 3C).

**Figure 4:**
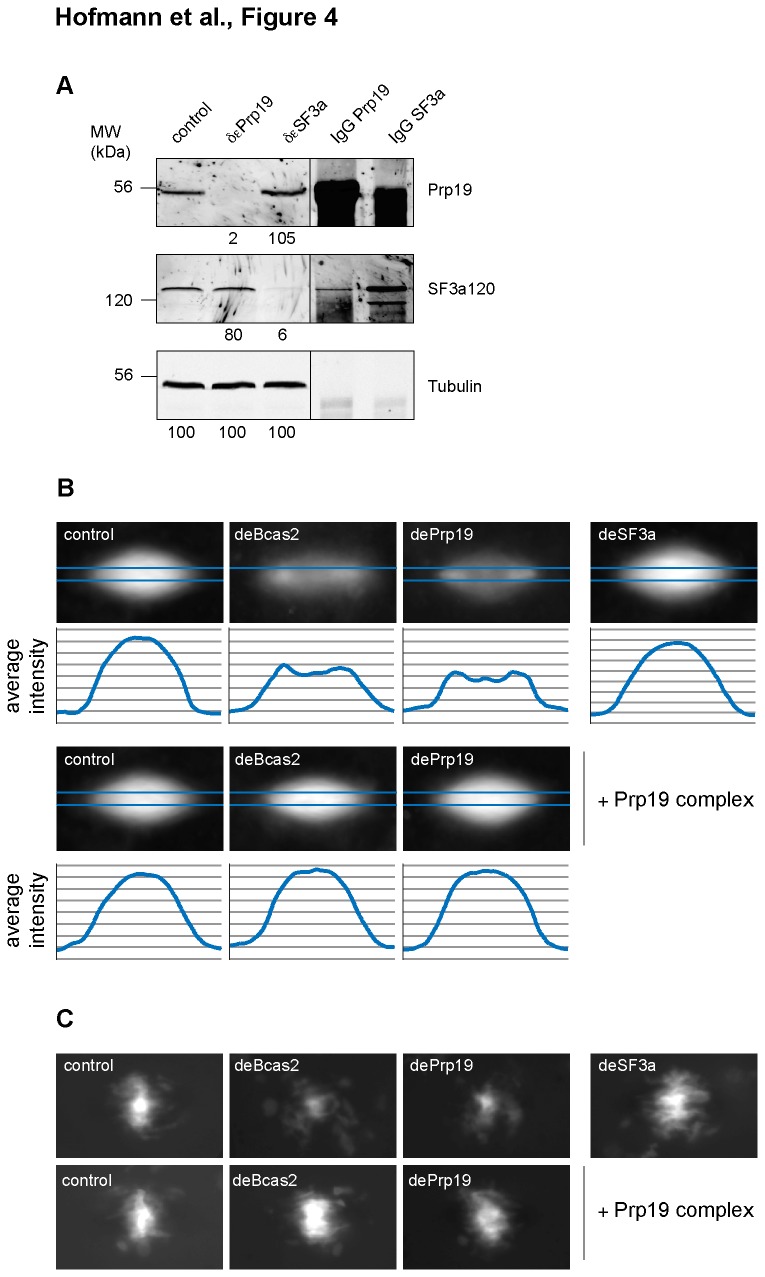
Depletion of the Prp19 complex specifically compromises spindle formation in *Xenopus* egg extracts. *Xenopus* egg extracts were depleted of the Prp19 complex or the U2 specific SF3a protein complex using specific antibodies (dePrp19, deSF3a120); an unspecific IgG served as a control. (A): The levels of Prp19 and the SF3a120 protein were analyzed by immunoblotting using tubulin as a loading control (see numbers). (B, C): Microtubule assembly was monitored in egg extracts after addition of sperm nuclei (DAPI, DNA, in C) and Cy3-tubulin (in B). 20 spindles were analyzed under all conditions, aligned and averaged (z-projection, average, ImageJ). (B): average microtubule intensity projections. Graphs show average intensity distributions along the pole-to-pole axes indicated in projections in blue. (C): chromatin distributions as determined from the DAPI signal.

To quantify the observed spindle defects, we compared the microtubule intensity distribution using overlays of 20-25 spindles under control conditions or after Prp19 complex depletion (see methods). We observed the previously described (Figures 2 and 3) characteristic reduction in microtubule density in the central part of the spindle after Prp19 complex depletion using antibodies against Bcas2 or Prp19 (Figure 4B). Importantly, add-back of the human purified Prp19 complex restored normal microtubule density distribution (Figure 4B, + Prp19 complex). Reduced microtubule density after Prp19 depletion led to a less efficient alignment of chromatin (Figure 4B, chromatin).

These results indicated that defects observed after Prp19 immunodepletion are only seen in spindles around sperm nuclei after an entire ex vivo cell cycle, i.e. after complete interphase. It seemed possible that residual mRNA mis-splicing events during the short interphase affected subsequent spindle formation in vitro. We therefore aimed to directly affect the splicing machinery by addition of the small molecule inhibitor of splicing, spliceostatinA (SSA [27]). SSA inhibits splicing of different mRNAs both in human cell free extracts and intact cells at a concentration of 60 nM by targeting proteins of the SF3b complex, which belong to the U2 snRNP specific proteins [27]. We tested mitotic spindle assembly in control and Prp19 complex-depleted extracts, after addition of high concentrations (500 nM) of SSA or AcSSA, a non-targeting, acetylated derivative of SSA. A significant number of spindles displayed low microtubule densities after Prp19 complex depletion, while no spindle abnormalities were observed after addition of SSA or AcSSA alone (Figure S2 A). Likewise, complete inhibition of transcription during the in vitro cell cycle using Actinomycin D left spindle assembly unaffected (Figure S2 B). This suggested that inhibition of splicing during interphase in vitro did not cause the observed mitotic spindle assembly defects. To confirm this hypothesis we directly immunodepleted the splicing-essential U2snRNP specific protein complex SF3a using an antibody against the largest subunit, SF3a120, and compared spindle formation in control extracts with Prp19 or SF3a depleted extracts (Figure 4A-C). Indeed, no differences between control spindles and spindles assembled after SF3a immunodepletion were observed (Figure 4B and C).

**Figure 5:**
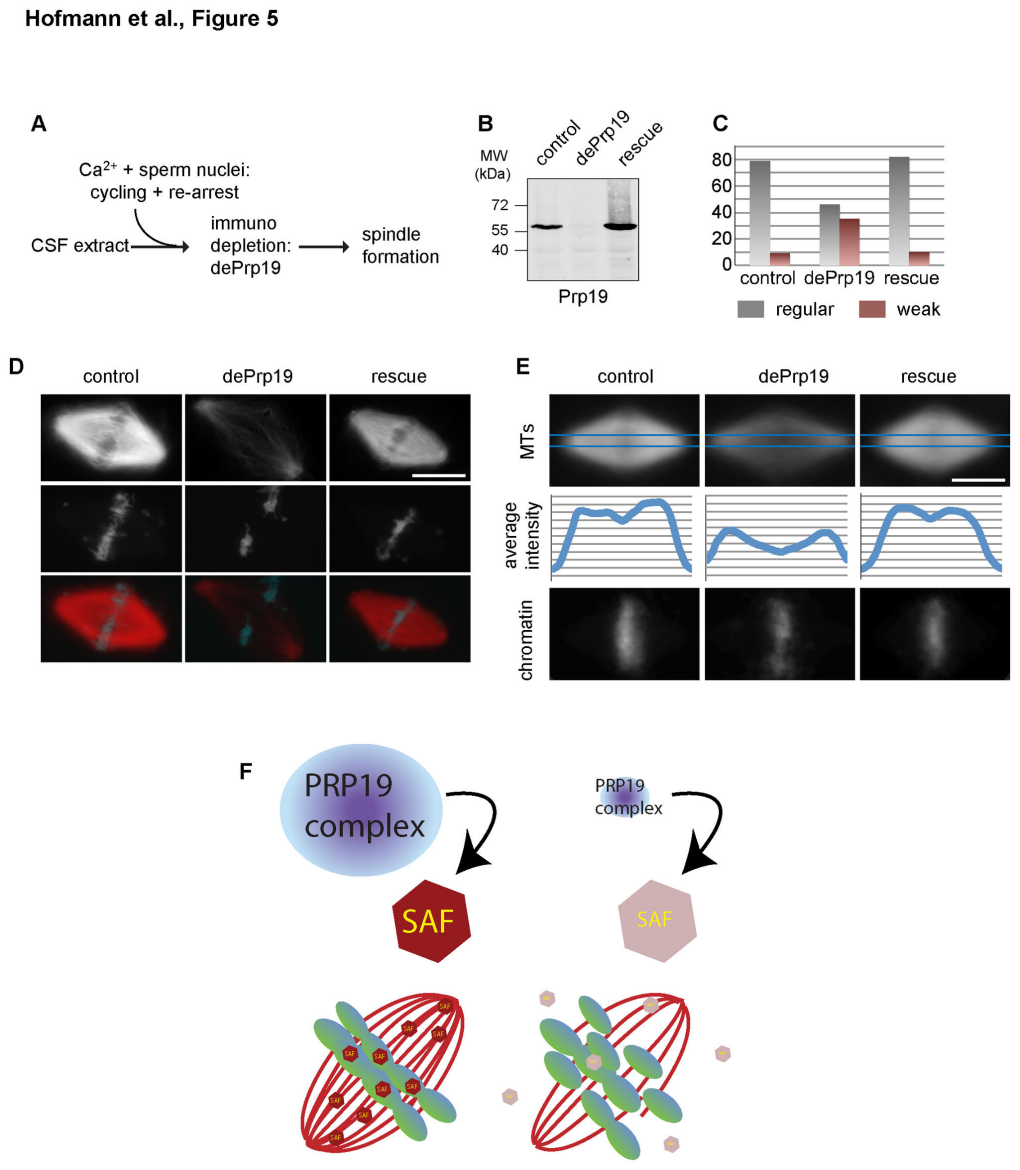
Immunodepletion of Prp19 directly in mitosis compromises spindle assembly. Spindle assembly was monitored using sperm nuclei in egg extracts after a complete cell cycle. Immunodepletion was performed only after rearrest in mitosis. (A): schematic overview of experimental setup. (B): Immunoblot to show depletion and rescue using a Prp19 specific antibody. (C): Quantification of normal (grey) and weak spindles (red). (D): Representative images of normal and weak spindles in control, depleted (dePrp19) and reconstituted extracts (rescue). (E): 30 spindles were analyzed under the conditions indicated, aligned and averaged (z-projection, average, ImageJ). upper panels (microtubules): average microtubule intensity projections. Graphs show average intensity distributions along the pole-to-pole axes indicated in projections in blue (middle panels). Lower panels: chromatin distributions as determined from the DAPI signal. Scale bars: 20 µm. (F): Model to explain the defects in spindle formation after Prp19 knock-down or depletion. Left: control situation: Prp19 modifies an unknown Spindle Assembly Factor (SAF), which directly contributes to spindle formation by modifying properties of microtubules as a microtubule associated protein, or working as a kinetochore-associated protein in stabilizing kinetochore to microtubule attachments. Right: after knock-down or immunodepletion of the Prp19 complex, the regulated SAF looses its function in spindle formation.

To further support our conclusion, we finally sought to temporally uncouple interphase functions from events in mitosis in the ex vivo cell cycle. We activated untreated CSF arrested egg extracts by calcium addition in the presence of sperm nuclei. These extracts regularly proceed through interphase and re-arrest in mitosis upon addition of fresh CSF extracts (Figure 5A). 10 minutes after re-addition of CSF extracts we cooled down the reactions, immunodepleted the Prp19 complex (Figure 5A and B) and further allowed spindle formation at 20°C after depletion. While spindles assembled normally in mock (control IgG) depleted extracts, immunodepletion of the Prp19 complex caused both a reduction in spindle formation, as well as an increased number of spindles with reduced microtubule density in the central part (Figure 5 C to E). Importantly, re-addition of the human Prp19 complex fully restored spindle formation (Figure 5 C to E, compare dePrp19 with rescue conditions). These data confirm a direct and specific function of the Prp19 complex in spindle formation and chromosome alignment, which is independent of the established role of the complex in splicing during interphase.

Taken together, we utilized *Xenopus* egg extracts to test a possible mitotic function of the Prp19 complex independent of its role in the interphase nucleus. *Xenopus* eggs are arrested in metaphase after a long phase of transcription, RNA processing and translation, during which the giant oocytes accumulate stockpiles of mature mRNAs and proteins. Although spindle localized protein production from endogenous mRNAs may play an important role in meiotic spindle assembly in intact oocytes [28], neither transcription (See Figure S2) nor translation of any messages, except of cyclin B, is required for spindle assembly in *Xenopus* egg extracts [22] [29].

Nevertheless, we consistently observed reduced microtubule density and frequent metaphase alignment problems upon immunodepletion of the Prp19 complex from *Xenopus* egg extracts. Several lines of evidence confirm the specificity of this observation. First, immunodepletion with two different antibodies against proteins of the Prp19 complex, Bcas2 and Prp19, yielded very similar results. Second, spindle assembly abnormalities were fully rescued upon addition of the intact Prp19 complex that was purified from human cells. Third, spindle assembly failures were not due to a generally reduced capacity of extracts to produce microtubules: abnormalities became apparent only in complete spindles containing replicated centrosomes and chromosomes, and paired sister kinetochores [24], but not in microtubule structures forming around chromatin beads, in RanGTP-mediated microtubule assemblies, or in non-cycled spindles (data not shown). Finally, the direct immunodepletion of the Prp19 complex from cycled, re-arrested *Xenopus* egg extracts qualitatively and quantitatively resulted in spindle assembly defects very similar to those observed upon depletion of the Prp19 complex during the entire cell cycle. Consistent with the loss of microtubule to kinetochore interactions in intact cells, our data from the *Xenopus* cell free system suggest that defects in spindle formation upon Prp19 depletion arise as a consequence of impaired microtubule to kinetochore attachments, which do not contribute to spindle formation in non-cycled spindles, chromatin beads or Ran structures [24,25]. Our data converge into a model, in which the Prp19 complex enables the function of a key spindle assembly factor (SAF, Figure 5F), which may directly contribute to kinetochore function, or regulate microtubule dynamics to allow proper attachment of microtubule plus ends to kinetochores (Figure 5F).

Taken together, our data clearly show essential functions of the Prp19 complex in mitotic spindle formation. Without a functional Prp19 complex, spindle assembly fails leading to improper chromosome alignment (Figure 5F). Although the exact mode of action of the Prp19 complex in mitosis remains elusive, our data identify Prp19 as the first spliceosome subcomplex that directly contributes to functions in open mitosis independent of its role in the interphase nucleus.

## Materials and Methods

### Antibodies

The following commercial antibodies were used: anti-BCAS2, Bethyl A300-915A; anti-CDC5L, Santa Cruz sc135863; anti-PLRG1, Bethyl A301-940A; anti SF3120/SaP114: Bethyl 00316; anti- α-Tubulin, Sigma T9026; HA, Covance clone 16B12; Aurora B; Abcam (ab2254); anti-NDC80, Abcam (ab33034); anti-CENPA, Abcam (ab13939). Antibodies against BCAS2 were generated, and sera affinity purified, using the following peptides: EEETRRYRPTKNYLS (identical in man and frog), NEIYQIKQQHGEANKENIRQ (human sequence) and NEVYQLKEQSGENKENIQDY (Frog sequence). Antibodies against Prp19 were generated, and sera affinity purified using the following peptides: TLRQQLQTTRQELS (identical in man and frog) and TERKKRGKTVPEELVKPEE(*D in X.l.*) LSKYR (identical apart from the indicated single amino acid substitution).

### siRNA oligonucleotides

siRNAs were synthetic double-stranded oligos from Life technologies. BCAS2: Stealth: HSS115723 (oligo 1), HSS115724 (oligo 2). Nuf2: Silencer Select: s37983.

### SDS-PAGE and immunoblotting

Proteins were separated by SDS-PAGE [30] and transferred onto nitrocellulose for imunoblotting [31]. To detect and quantify secondary antibody signals, the LI-COR Odyssey system was used.

### Cell culture and transfection

HeLa CCL2 cells were grown in high glucose Dulbecco’s modified Eagle’s medium (DMEM; Invitrogen) supplemented with 10% fetal calf serum (FCS), 2 mM L-glutamine, 100 µg/ml penicillin and streptomycin at 37°C in a humidified 5% CO_2_ incubator. Transient transfection of HeLa cells with plasmid DNA was performed with Lipofectamine 2000 Transfection Reagent (Life technologies) following the manufacturer’s recommendations. Transient transfection of cells with RNAi oligonucleotides was performed with LipofectamineRNAi Max Transfection Reagent (Life technologies) following the manufacturer’s recommendations. Final concentrations of 50 nM (stealth) or 20 nM (silencer select) siRNA oligonucleotide were used.

### Sample preparation and imaging after immunofluorescence

For immunofluorescence microscopy, HeLa cells were grown on 0.01% poly-L-lysine (SIGMA)-coated coverslips and fixed in methanol at -20 °C for 5 min. Analysis and imaging was performed on an Olympus, Delta Vision fluorescence microscope equipped with an Olympus 100/1.4 NA Oil objective using the Soft Worx software, or an LSM780 confocal microscope using a Plan-APOCHROMAT 63x/1.4 Oil objective and the Zeiss ZEN 2010 software. Image processing was performed with ImageJ analysis software (Soft Imaging System) and Adobe Photoshop CS5. For the analysis of microtubule end-on attachments to kinetochores, mitotic cells were recorded using the Delta vision system. Attachments were determined visually and quantified from 60 k-fibers in each of 3 independent experiments. Interkinetochor-distances were determined using the corresponding pairwise CENPA signals in the same images.

### Quantitative evaluation of spindle morphology

Analysis and imaging of *Xenopus* spindles was performed with a Olympus IX81 inverted microscope with PLAPO 100/1.45 NA Oil objective and the Cell R software. To measure microtubule intensity in spindles, images were rotated to align the pole to pole axis horizontally and afterwards cropped using fixed sized rectangulars in Adobe Photoshop CS5. Using the ImageJ64 1.45s analysis software (Soft Imaging System) cropped files were defined as stacks and overlaid as z-projections (average intensity).

### Time Lapse Imaging of Human Cells

HeLa Kyoto cells stably expressing mCherry-Histone2B were used for time lapse experiments. Cells were plated on µ-slide 8 well ibi, Treat chambers (ibidi) and imaged for 48h on an Olympus CellR system in 20 min. intervals. We used an Olympus IX81 inverted microscope with a UPLSAPO 20x/0.75 air objective and a Hamamatsu ORCA-R2 camera and the Olympus excellence software for image acquisition. Cells were kept in DMEM/FCS at 37°C and 5% CO2 during imaging.

### Preparation of *Xenopus laevis* egg extracts

CSF-arrested (M-Phase) *X. laevis* egg extracts were prepared as described previously (Murray, 1991).

### Spindle assembly in *Xenopus* egg extracts

Spindle assembly was induced by adding Cy3-labelled tubulin, energy mix (5 mM creatine phosphate, 25 µl/ml creatine kinase, 0.25 mM ATP and GTP) and sperm nuclei (500 nuclei/µl) to CSF extracts. To assemble the spindles, the extract was released to interphase by the addition of 0.6 mM CaCl_2_ for 60-90 min and cycled back into M-phase by the addition of one volume of fresh CSF extract.

After 45 to 60 min, spindles were fixed in 1 ml BRB80 (80 mM K-PIPES, pH 6.8, 1 mM EGTA, 1 mM MgCl_2_) containing 30% glycerol, 0.25% glutaraldehyde, and 0.1% Triton X-100, and subsequently centrifuged (HB4 rotor, 12,000 rpm, 12 min, 20°C) through a 5 ml 40% glycerol cushion in BRB80 onto poly-L-lysine–coated coverslips as described by 32. Chromatin bead spindles were assembled as described [33].

### Ran-induced microtubule assembly

Recombinant Ran in its GTP-bound form (RanQ69L, 20 µM, preloaded with GTP) and Cy3-labelled tubulin were added to CSF-arrested extracts. After an incubation of about 20 min at 20°C, samples were fixed and squashed [24] with 1 volume of fix solution (ratios: 0.3 ml formaldehyde 37%, 0.6 ml 80% glycerol, 1 ml 1xMMR and 1 µl 10 mg/ml Hoechst).

### Immunodepletion, complementation and inhibitors in *Xenopus* egg extracts

For immunodepletions, 25 µg of affinity-purified X.l.Bcas2 or Prp19 antibody was bound to 50 µL of protein A–conjugated Dynabeads (Life Technologies) and resuspended in 150 µl of CSF-extract. As a control, a non-specific IgG antibody was coupled to the same amount of beads as used in the experiment. For rescue experiments purified, HA-Flag-tagged human Prp19 complex [21] or GFP-tagged human BCAS2 were added to the depleted extract before the spindle assembly reaction at a concentration of 100 nM. SSA or AcSSA were used at a final concentration of 500 nM. Actinomycin D was used at a concentration of 5 µg/µl (4 mM).

## Supporting Information

Figure S1 **Localization of Prp19 complex proteins.** (A): Localization of BCAS2 as determined by indirect immunofluorescence after knockdown (siRNA BCAS2) of human BCAS and reexpression using the *Xenopus* (X.l.) BCAS2 ortholog. (B): Co-regulation of BCAS2 (red) and PLRG1 (left, green) or PRPF19 (right, green) upon knock-down and rescue of BCAS2 as shown in (A). (C): PRPF19 (green in merge) and tubulin (red in merge); (D): CDC5L (green in merge), and PLRG1 (red in merge) were visualized by indirect immunofluorescence together with DAPI (blue in merge) to stain the DNA in Interphase (I) and Mitosis (M). Scalebars: 10 µm.
(TIF)

Figure S2 **Addition of transcription or splicing inhibitors to *Xenopus* egg extracts does not lead to mitotic abnormalities.** (A): Spindle assembly was monitored in control or Prp19 complex (deBCAS2) depleted egg extracts in the presence of SSA or AcSSA after a complete cell cycle. Intact spindles were counted in 20 µl assembly reactions. The graph shows mean values from three independent experiments +/- s.e.m. (B): Spindle assembly was monitored in control or Prp19 complex (deBCAS2) depleted egg extracts in the absence or presence of Actinomycin D (ActD) after a complete cell cycle. Intact spindles were counted in a 20 µl assembly reaction.
(TIF)

Movie S1 **Time-lapse recordings of control human cells.** Cells stably expressing histone 2B-GFP that were monitored for 48 hours, 66 hours after siRNA transfection, in 30 min. time intervals using an Olympus IX81 inverted microscope equipped with a 20 x objective.
(MOV)

Movie S2 **Time-lapse recordings of human cells after BCAS2 knock-down.** Cells stably expressing histone 2B-GFP that were monitored for 48 hours, 66 hours after siRNA transfection, in 30 min. time intervals using an Olympus IX81 inverted microscope equipped with a 20 x objective.
(MOV)
