# Supplementary figures and images for: The Prp19 Complex Directly Functions in Mitotic Spindle Assembly
Source: PLoS One. 2013 Sep 19;8(9):e74851. doi: 10.1371/journal.pone.0074851 (PMC3777999; doi:10.1371/journal.pone.0074851)

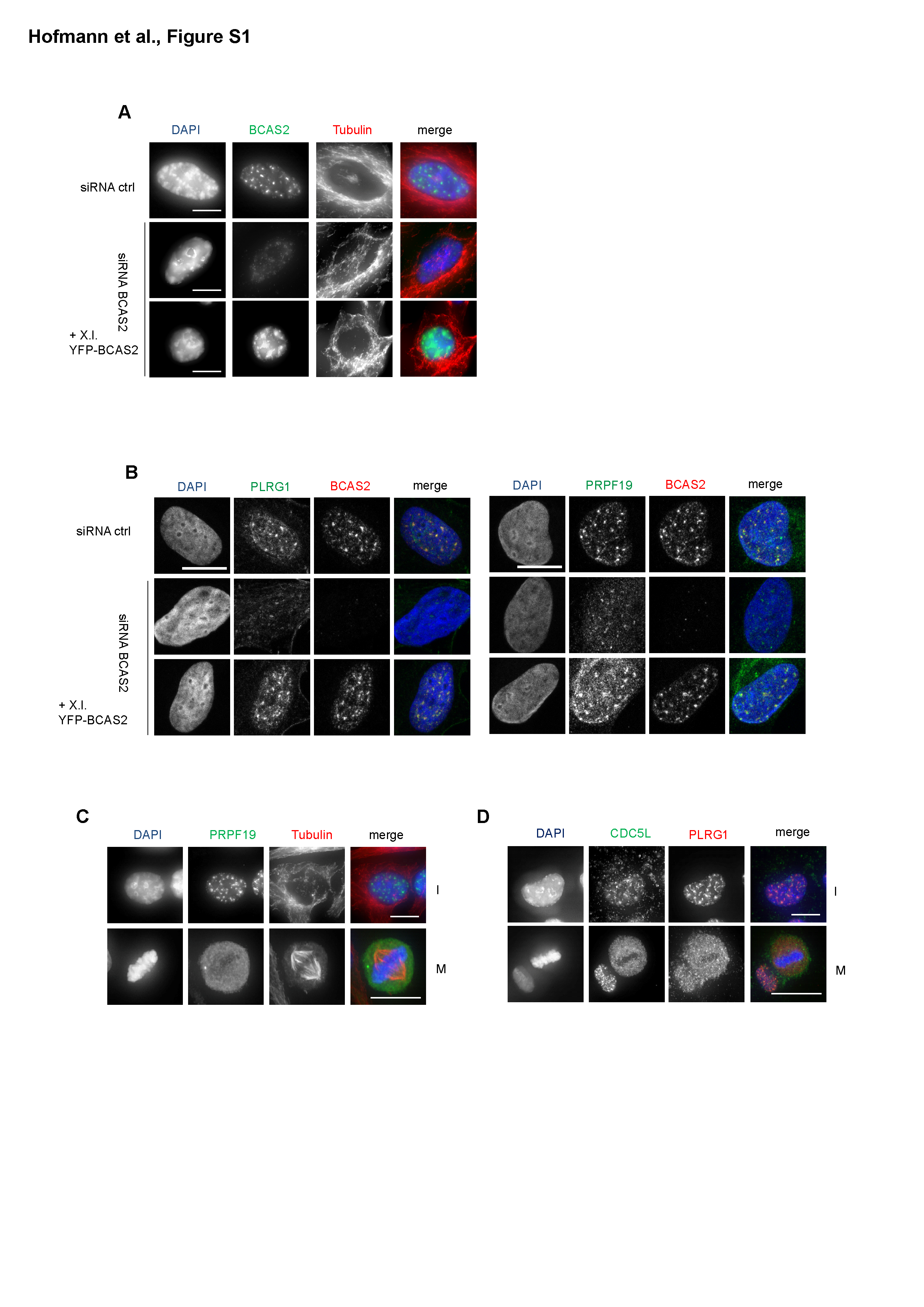

Supplement: Figure S1 — Localization of Prp19 complex proteins. (A): Localization of BCAS2 as determined by indirect immunofluorescence after knockdown (siRNA BCAS2) of human BCAS and reexpression using the Xenopus (X.l.) BCAS2 ortholog. (B): Co-regulation of BCAS2 (red) and PLRG1 (left, green) or PRPF19 (right, green) upon knock-down and rescue of BCAS2 as shown in (A). (C): PRPF19 (green in merge) and tubulin (red in merge); (D): CDC5L (green in merge), and PLRG1 (red in merge) were visualized by indirect immunofluorescence together with DAPI (blue in merge) to stain the DNA in Interphase (I) and Mitosis (M). Scalebars: 10 µm. (TIF) [file pone.0074851.s001.tif]

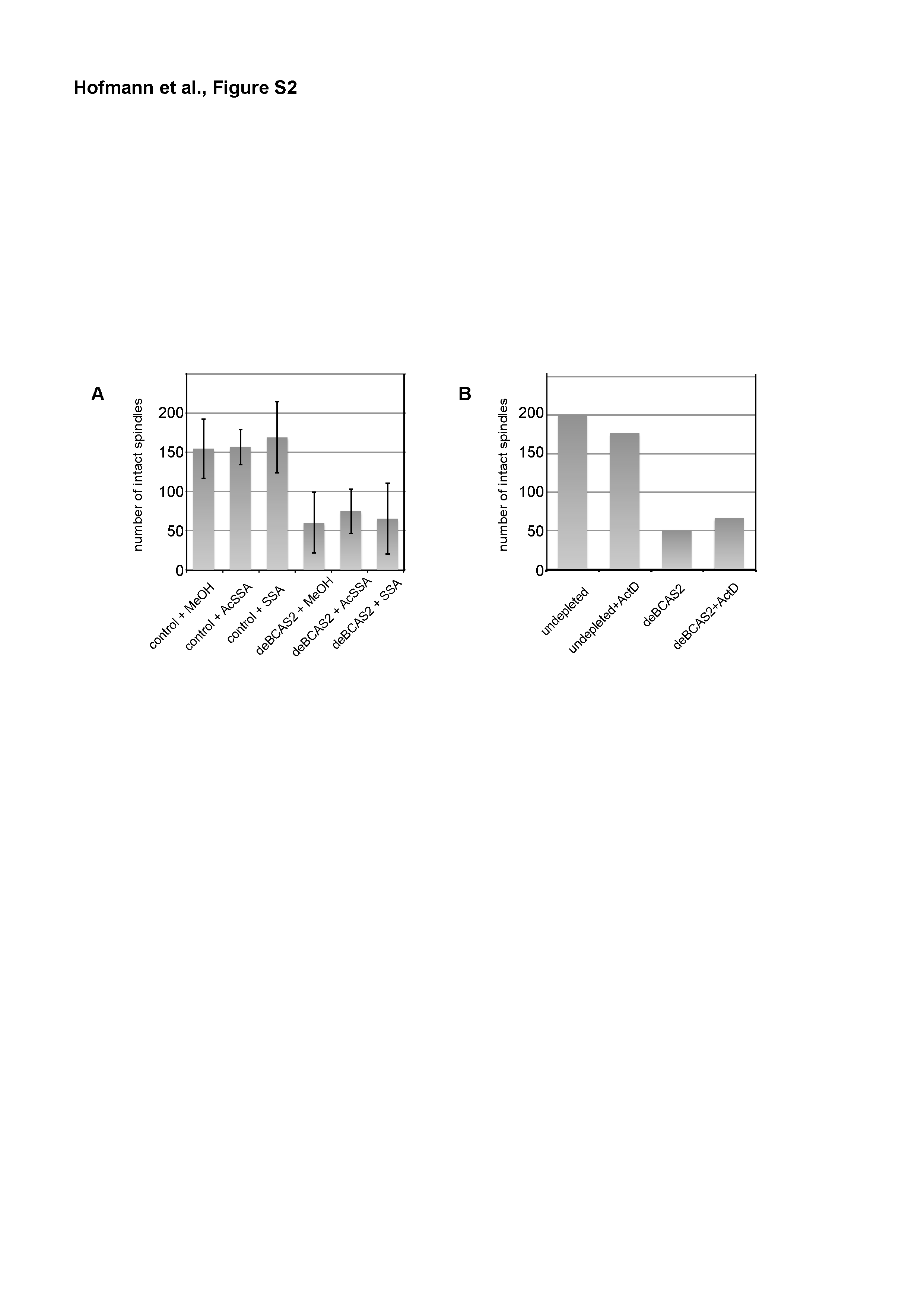

Supplement: Figure S2 — Addition of transcription or splicing inhibitors to Xenopus egg extracts does not lead to mitotic abnormalities. (A): Spindle assembly was monitored in control or Prp19 complex (deBCAS2) depleted egg extracts in the presence of SSA or AcSSA after a complete cell cycle. Intact spindles were counted in 20 µl assembly reactions. The graph shows mean values from three independent experiments +/- s.e.m. (B): Spindle assembly was monitored in control or Prp19 complex (deBCAS2) depleted egg extracts in the absence or presence of Actinomycin D (ActD) after a complete cell cycle. Intact spindles were counted in a 20 µl assembly reaction. (TIF) [file pone.0074851.s002.tif]
